# ECMO decannulation is associated with dynamic changes in coagulation profiles: an exploratory, nested cohort study

**DOI:** 10.1186/s12871-026-03641-1

**Published:** 2026-01-29

**Authors:** Matthias Noitz, Dennis Jenner, Roxane Brooks, Johannes Szasz, Romana Erblich, Bernhard Eichler, Marius Knöll, Niklas Krenner, Tina Tomić-Mahečić, Martin W. Dünser, Andreas Zierer, Jens Meier

**Affiliations:** 1 Department of Anesthesiology and Critical Care Medicine, Kepler University Hospital GmbH, Johannes Kepler University Linz, Krankenhausstraße 9, 4020 Linz and Altenberger Strasse 69, Linz, 4040 Austria; 2 Medical Faculty, Johannes Kepler University, Linz, Austria; 3 Department of Cardiothoracic and Vascular Surgery, Kepler University Hospital GmbH, Johannes Kepler University Linz, Krankenhausstraße 9, 4020 Linz and Altenberger Strasse 69, Linz, Linz, 4040 Austria; 4 Department of Anaesthesiology and Intensive Care Medicine, University Hospital Center Zagreb - Rebro, Zagreb, Croatia

**Keywords:** Coagulation, Extracorporeal membrane oxygenation, ECMO, Thrombelastometry, Factor XIII

## Abstract

**Background:**

ECMO-associated coagulopathy is a common phenomenon in patients undergoing ECMO therapy. However, data on coagulation trajectories in the post ECMO decannulation period are limited. This study aimed to explore changes in coagulatory function within 72 h after weaning of ECMO therapy.

**Methods:**

Exploratory, nested cohort study of a prospective, observational, single-centre cohort study investigating haemostatic changes in adult patients undergoing ECMO therapy at a tertiary academic centre and ECMO referral facility in Linz/Austria. Coagulation tests as well as ROTEM^®^ analyses were performed at the time of ECMO decannulation (d0) and three days later (d3). Intra-individual changes in coagulation parameters and viscoelastic test results after weaning of ECMO therapy were determined using paired comparisons (Student’s *t*-tests or Wilcoxon signed-rank tests) between the two timepoints.

**Results:**

30 adult patients were included into the final analysis. Platelet counts [97 (82–149) vs. 211 (161–346) G/L, *p* < 0.001)], Factor VIII activity [294 (208–427) vs. 400 (304–450) %, *p* = 0.03] and Factor XIII activity [63 (52–73) vs. 86 (70–96) %, *p* < 0.001] increased significantly between d0 and d3. Fibrinogen levels trended upwards after decannulation [601 (419–711) vs. 650 (495–790) mg/dL, *p* = 0.08]. Antithrombin activity [96 (83–117) vs. 111 (93–130) %, *p* = 0.001], Protein C [83 (69–99) vs. 93 (90–104) %, *p* = 0.01] and Protein S activity [78 (60–91) vs. 80 (72–103), *p* = 0.002] significantly increased between the two timepoints. D-Dimer levels significantly declined between d0 and d3 [13.5 [6–29] vs. 8.1 (3.9–17.3) %, *p* = 0.019]. ROTEM^®^ analyses showed significant decreases in INTEM^®^ coagulation time and clot formation time, and significant increases in clot amplitude at five and ten minutes, as well as maximum clot firmness in the INTEM^®^ and EXTEM^®^ assays between d0 and d3.

**Conclusions:**

Extensive changes in coagulation profiles were observed within 72 hours following ECMO weaning and decannulation. These changes reflect a rapid restoration of haemostasis and possibly an early shift towards a prothrombotic phenotype.

**Supplementary Information:**

The online version contains supplementary material available at 10.1186/s12871-026-03641-1.

## Introduction

Extracorporeal membrane oxygenation (ECMO) offers potentially life-saving support for patients with severe respiratory or circulatory failure [1–5]. Despite its benefits, ECMO carries substantial risks including bleeding, haemolysis, infection, as well as thromboembolic, neurologic and renal complications [6–11]. Major bleeding and thromboembolic events occur in 30–60% of patients, relevantly contributing to morbidity and mortality of patients undergoing ECMO therapy [12–15].

ECMO-associated coagulopathy reflects a complex, multifactorial disorder of the coagulation system driven by the cause of critical illness (e.g., cardiac surgery), systemic inflammation and coagulation activation induced by interactions between blood and artificial surfaces of the ECMO circuit, altered blood flow kinetics, device-related shear stress, and anticoagulation [16–18]. ECMO-associated coagulopathy typically results in thrombocytopenia, platelet dysfunction, acquired von Willebrand syndrome, “waste coagulopathy“ with consumption of coagulation factors (e.g., hypofibrinogenemia and acquired factor XIII deficiency) and dysregulated fibrinolysis [19–23].

While most research around ECMO-associated coagulopathy has so far focused on coagulation abnormalities during ECMO therapy, the trajectory of ECMO-associated coagulopathy following weaning of ECMO therapy is less well characterized. Observational data both in children and adults suggest that ECMO-associated coagulopathy reverses within hours to a few days after decannulation [24–26]. Limited evidence even suggests that an overshooting reversal of ECMO-associated coagulopathy may translate into a transient hypercoagulable state following weaning of ECMO therapy [20, 26]. These preliminary data have given rise to the hypothesis that ECMO therapy is not only associated with relevant coagulopathy following its initiation but also its termination.

This exploratory study aims to analyse changes in coagulatory function within 72 h after weaning of ECMO therapy in 30 adult patients.

## Methods

This analysis was designed as an exploratory, nested cohort study of a prospective, observational, single-centre cohort study investigating haemostatic changes in adult patients undergoing ECMO therapy [27]. It was conducted in a 22-bed intensive care unit at the Department of Anesthesiology and Critical Care Medicine, Kepler University Hospital, in Linz/Austria between December 01, 2022 and October 31, 2024. The study site is a tertiary academic centre and ELSO (Extracorporeal Life Support Organization)-certified ECMO referral facility. The study protocol was reviewed and approved by the local Medical Ethics Committee of the Medical Faculty of the Johannes Kepler University Linz (reference number: 1008/2022; approved on July 12, 2022). Written informed consent was obtained from all study patients or their next of kin. This manuscript was drafted in accordance with the STROBE (Strengthening the Reporting of Observational Studies in Epidemiology) checklist for cohort studies [28].

### Study population

The primary study included 44 adult (≥ 18 years) patients without known hereditary haemorrhagic or thrombophilic disorders, who underwent ECMO therapy (> 24 h) at the study centre during the observation period. Only patients who were successfully weaned off ECMO support were eligible for enrolment in this nested cohort study. Exclusion criteria were the use of the dual-pump CARL^®^ system (Controlled Automated Reperfusion of the Whole Body, RESUSCITEC GmbH, Freiburg, Germany), ECMO use for temporary right ventricular support following implantation of a left ventricular assist device, and ECMO use as a bridge to another mechanical circulatory assist device.

### ECMO management

The institutional protocols for the care of patients on ECMO support as well as anticoagulation and haemostatic management during ECMO therapy have been published before [27, 29]. Briefly, ECMO support was provided with the Xenios Console^®^ platform (Xenios AG, Fresenius Medical Care, Heilbronn, Germany), heparin–albumin–coated tubing (Novalung XLung kit 230^®^; Xenios AG, Fresenius Medical Care), and heparin–albumin–coated vascular cannulas (Getinge AB^®^, Gothenburg, Sweden). Unfractionated heparin was the first-line anticoagulant, administered as a continuous infusion and titrated to an activated partial thromboplastin time target of 50–60 s. Additionally, epoprostenol was continuously infused into the oxygenator (0.005–0.01 mg per hour) to mitigate platelet activation, according to the local anticoagulation protocol. Anticoagulation therapy was withheld 3–4 h before end of ECMO support and decannulation. In patients on veno-venous ECMO support, decannulation was performed by the critical care team at the bedside using skin sutures with both manual compression and pneumatic compression dressings (Safeguard^®^; Datascope Corporation, Fairfield, New Jersey, United States) for insertion site wound closure. Decannulation in patients on veno-arterial ECMO support was performed surgically with cannula removal, vessel ligation and incision closure by the cardiovascular surgery team. Prophylactic anticoagulation with low-molecular-weight heparin was initiated within 24 h after decannulation.

### Study variables and data collection

The following study-related variables were collected in all subjects: age, sex, body mass index, comorbid conditions, Simplified Acute Physiology Score II [30] at ICU admission, indications for ECMO therapy, ECMO configuration, cannula size, details of the ECMO run (maximum blood flow rate, maximum sweep gas flow rate, maximum pump speed, minimum pre-membrane pressure, maximum post-membrane pressure, maximum pressure-gradient), mode of anticoagulation, duration of the ECMO run, type and dose of anticoagulation therapy, number of blood products transfused and coagulation products administered during 72 h following decannulation, intensive care unit length of stay, as well as all-cause mortality at intensive care unit and hospital discharge. In addition, baseline coagulation parameters prior to ECMO initiation were collected.

All study-specific coagulation tests were performed at the time of decannulation (d0) and three days later (d3). At these pre-specified timepoints, the following coagulation tests were performed: platelet count, activated partial thromboplastin time, prothrombin time, anti-Xa levels calibrated for unfractionated heparin and low-molecular-weight heparin, fibrinogen concentrations, Factor VIII activity, Factor XIII activity, antithrombin activity, protein C activity, protein S activity, and D-dimer levels. Thrombocytopenia was defined as a platelet count < 150 G/L, hypofibrinogenemia as fibrinogen concentrations < 150 mg/dL, as well as Factor VIII and XIII deficiencies as a factor activity < 70%. All laboratory analyses were conducted in the institutional central laboratory of the study centre using calibrated automated analysers and manufacturer-recommended reagents according to internal standard operating procedures and external quality control standards. Fibrinogen levels were measured based on the Clauss method and coagulation factor XIII activity was measured using a chromogenic assay (Berichrom^®^ FXIII chromogenic assay; Siemens Healthineers, Marburg, Germany), performed on a Sysmex CN-6000 analyzer. At the same timepoints (d0 and d3), viscoelastic coagulation tests were conducted using the fully automated point-of-care ROTEM^®^ sigma device (Werfen, Barcelona, Spain) [31, 32]. Critical care physicians instructed in the use of point-of-care thrombelastometry performed all measurements within one hour after blood sampling. ROTEM^®^ measurements included the following four assays: EXTEM^®^ (recombinant tissue factor-activated assay reflecting the extrinsic coagulation pathway), INTEM^®^ (ellagic acid-activated assay reflecting the intrinsic coagulation pathway), FIBTEM^®^ (cytochalasin D mediated inhibition of platelet activity) and APTEM^®^ (addition of aprotinin to inhibit fibrinolysis). Within each assay, the following coagulation parameters were determined: clotting time (CT), clot formation time (CFT), clot amplitude at 5 and 10 min respectively (A5 and A10), maximum clot firmness (MCF), as well as lysis indexes at 60 min (LI 60).

### Study goals and outcomes

The goal of this study was to report changes in platelet counts and the incidence of thrombocytopenia, changes in activated partial thromboplastin time, prothrombin time, anti-Xa levels, D-Dimer levels, procoagulatory (fibrinogen, Factor VIII activity, Factor XIII activity) and anticoagulatory factors (antithrombin, Protein C activity, Protein S activity) as well as viscoelastic coagulation test results during the first 72 h following ECMO weaning and decannulation.

### Statistical analysis

All statistical analyses were performed using the R statistical software package (Version 4.4.3; R Core Development Team, Vienna, Austria) employing the rstatix, ggpubr, and ggplot2 packages. Before statistical analysis, all data were reviewed for plausibility and completeness. Implausible values and extreme outliers were excluded and corrected whenever possible. No imputation method was used to compensate for missing data. Normality of distribution of continuous variables was assessed using Shapiro–Wilk tests, histograms, and Q–Q plots. Categorical variables were presented as absolute frequencies and percentages. Continuous variables were summarized as median values with interquartile ranges (IQRs). Intra-individual changes in coagulation parameters and viscoelastic test results after weaning of ECMO therapy were determined using paired comparisons between the two timepoints (d0 and d3) on complete pairs per marker. Depending on normality distribution, within-patient differences were analysed using paired Student’s *t*-tests (in case of normally distributed variables) or Wilcoxon signed-rank tests (in case of non-normally distributed variables), with effect sizes reported as Cohen’s d_z_ or rank-biserial r. McNemar test and matched Odds ratios were used to compare paired binary outcome measures using continuity correction or exact binomial when discordant counts where < 10. A two-sided *p*-value < 0.05 was considered to indicate statistical significance. No corrections for multiple testing were applied due to the exploratory nature of the study. Sensitivity analyses were performed for coagulation parameters comparing day 0 and day 3, excluding patients who received blood transfusions or coagulation factor products during this period to minimize treatment-related bias.

## Results

Of the 44 patients included in the primary study, 32 subjects could be weaned off ECMO and decannulated. Two patients were excluded because of missing study data. Thirty patients (30 ECMO runs) were included into the statistical analysis. No patient was lost to follow-up. Demographic, clinical and ECMO-related data of the study population are summarized in Table 1. Surgical and perioperative characteristics of patients undergoing surgery prior to ECMO initiation are provided in Electronic Supplementary Table S1.

**Table 1:** Demographic, clinical and ECMO characteristics of the study population

*n*		30
Age	*years*	63.0 (50.5–68.5)
Male sex	*n* (%)	23 (76.7)
Body mass index	*kg/m²*	27.0 (24.4–29.0)
Comorbid conditions		
*arterial hypertension*	*n* (%)	11 (36.7)
*dyslipidaemia*	*n* (%)	11 (36.7)
*coronary artery disease*	*n* (%)	10 (33.3)
*cardiomyopathy*	*n* (%)	9 (30.3)
*diabetes mellitus*	*n* (%)	6 (20.0)
*chronic respiratory disease*	*n* (%)	4 (13.3)
*Immunosuppression*	*n* (%)	4 (13.3)
*chronic liver disease*	*n* (%)	3 (10.0)
*chronic kidney disease*	*n* (%)	3 (10.0)
SAPS II Score*	*points*	45 (39–59)
ECMO configuration		
*veno-arterial*	*n* (%)	24 (80)
*veno-venous*	*n* (%)	6 (20)
Indications for veno-arterial ECMO support (*n* = 24)		
*postcardiotomy LCOS*	*n* (%)	10 (41.7)
*ECPR*	*n* (%)	6 (25)
*cardiogenic shock*	*n* (%)	6 (25)
*septic shock*	*n (%)*	1 (4.2)
*right heart failure*	*n* (%)	1 (4.2)
Indications for veno-venous ECMO support (*n* = 6)		
*secondary ARDS*	*n* (%)	4 (66.7)
*pneumonia*	*n* (%)	1 (16.7)
*hypercapnic respiratory failure*	*n* (%)	1 (16.7)
ECMO-related data		
*arterial cannula size*	*French*	19 (18–19)
*venous cannula size*	*French*	25 (25–25)
*maximum blood flow*	*L/min*	4.6 (4.2–5.1)
*maximum sweep gas flow*	*L/min*	3.0 (2.5-4.0)
*maximum pump speed*	*rpm*	7,700 (7,400-8,100)
*minimum pre-membrane pressure*	*mmHg*	−43 (−60 − −27)
*maximum post-membrane pressure*	*mmHg*	299 (270–325)
*maximum pressure drop*	*mmHg*	71 (57–86)
Baseline haemostatic profile prior to ECMO initiation		
*FXIII*	%	70 (57–73)
*Fibrinogen*	mg/dL	357 (243–506)
*Platelets*	G/L	155 (112–191)
*AT*	%	69 (61–99)
*D-Dimer*	µg/mL	4.05 (1.2–5.76)
*aPTT*	seconds	27.5 (25.2–32.4)
*PT*	%	81 (69–100)
Anticoagulation during ECMO		
*unfractionated heparin*	*n (%)*	30 (100)
*unfractionated heparin (hourly dose)*	*IU/kg/h*	8.5 (5.3–12.1)
*additional epoprostenol*	*n (%)*	30 (100)
*anticoagulation switch to argatroban*	*n (%)*	2 (6.7)
Duration of ECMO run	*days*	8.0 (5.5–11.0)

*, calculated based on most aberrant values collected during the first 24 h after intensive care unit admission

*aPTT* activated partial thromboplastin time, *ARDS* acute respiratory distress syndrome, *AT* antithrombin, *ECMO* extracorporeal membrane oxygenation, *ECPR* extracorporeal cardiopulmonary resuscitation,* FXIII* coagulation factor XIII, *LCOS* low cardiac output syndrome,* PT* prothrombin time, *SAPS* Simplified Acute Physiology Score, *VA* veno-arterial; VV, veno-venous

Data are given as median values with interquartile ranges in parentheses, if not otherwise indicated

Twenty-one (70%) patients (95% CI 50.6–85.3) required pRBC transfusion [median number: 2 (IQR 1–4) units] within the first 72 h following decannulation. Fresh frozen plasma was transfused in 12 (40.0%) patients (95% CI 22.7–59.4) prior to ECMO decannulation, and in 3 (10.0%) of patients (95% CI 2.1–26.5) [median number: 2 (IQR 2–2) units] between d0 and d3. Platelet concentrate transfusion occurred in 22 (73.3%) patients (95% CI 54.1–87.7) during ECMO therapy, and in 5 (16.7%) study patients (95% CI: 5.6–34.7%) [median number: 1 (IQR 1–2) unit], after ECMO decannulation respectively. Additionally, 14 (46.7%) patients (95% CI 28.3–65.7) received fibrinogen concentrate substitution prior to decannulation, while only 1 (3.3%) patient (95% CI: 0.1–17.2%) received fibrinogen concentrate between d0 and d3. FXIII concentrates were administered in 17 (56.7%) patients (95% CI 37.4–74.5) with the last dose given a median of 2 (IQR 2–4) days before decannulation, at a median dose of 1,250 IU (IQR 1,250–2,500). In addition, 8 (26.7%) study patients (95% CI 12.3–45.9%) were treated with FXIII concentrates [median dose: 1,875 (IQR 1,250–2,500) IU] post decannulation. 7 (23.3%) of patients (95% CI 9.4–42.3) were treated with prothrombin complex concentrates during their ECMO run. No study patient received prothrombin complex concentrates between d0 and d3. In 8 (26.7%) patients (95% CI 12.3–45.9%), therapeutic anticoagulation with low-molecular-weight heparin (enoxaparin) was initiated within 72 h following decannulation. Twenty-one (70%) study patients (95% CI 50.6–85.3%) received low-molecular-weight heparin in weight-adjusted prophylactic dose, and 1 (3.3%) patient (95% CI 0.1–17.2) received no anticoagulation because of bleeding complications post decannulation. The median intensive care unit length of stay of study patients was 15.0 (12.2–23.8) days. Three patients (10%) died during their intensive care unit stay. All-cause hospital mortality of decannulated patients was 13.3% (4/30).

### Changes in platelet count and coagulation parameters

**Fig. 1:**
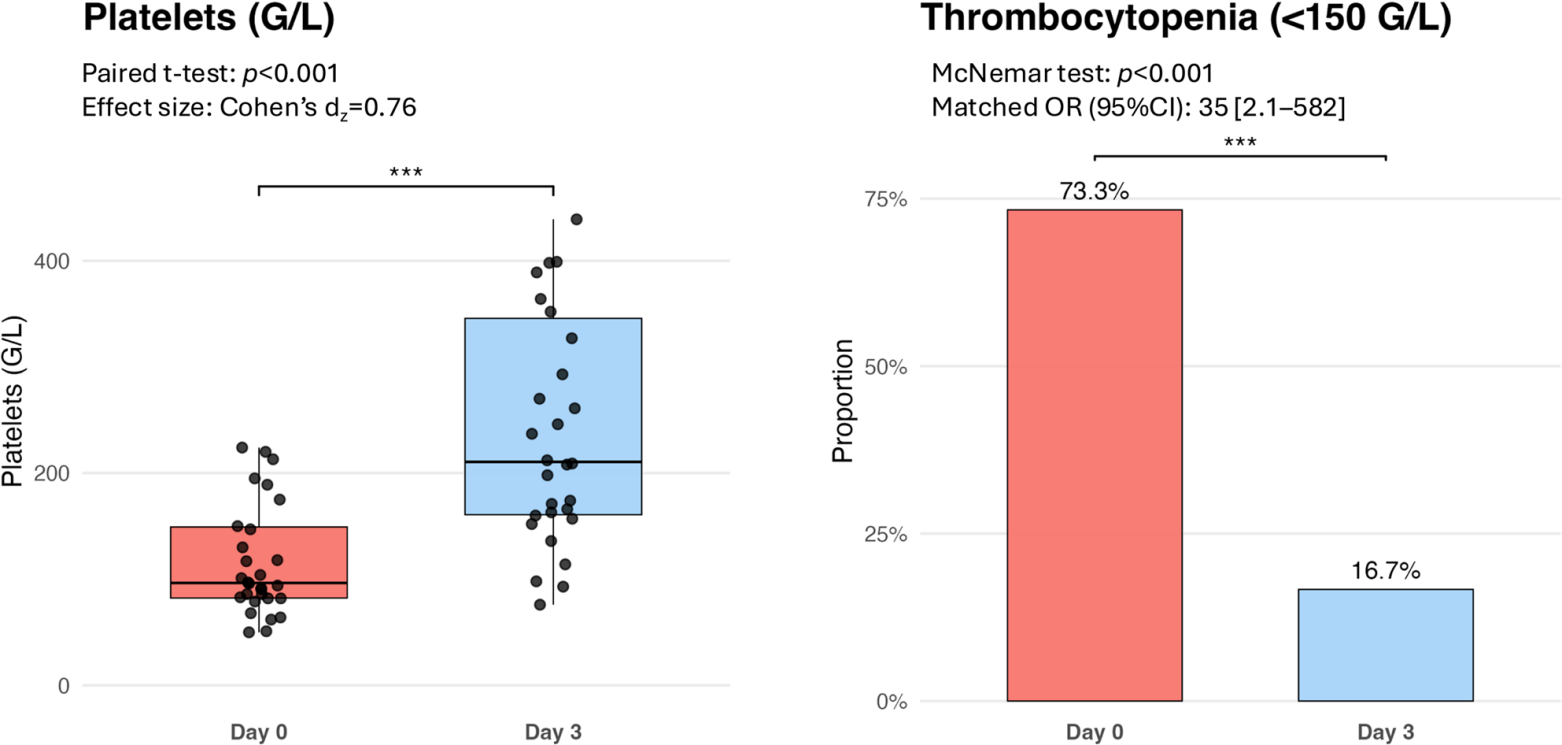
Changes in platelet count and prevalence of thrombocytopenia following ECMO decannulation. Left panel: Boxplot showing paired platelet counts on day 0 (immediately before decannulation) and day 3 after ECMO discontinuation. Data are displayed as medians with interquartile ranges and individual patient values (dots). Right panel: Bar graph showing the proportion of patients with thrombocytopenia (< 150 G/L) at both time points

**Fig. 2:**
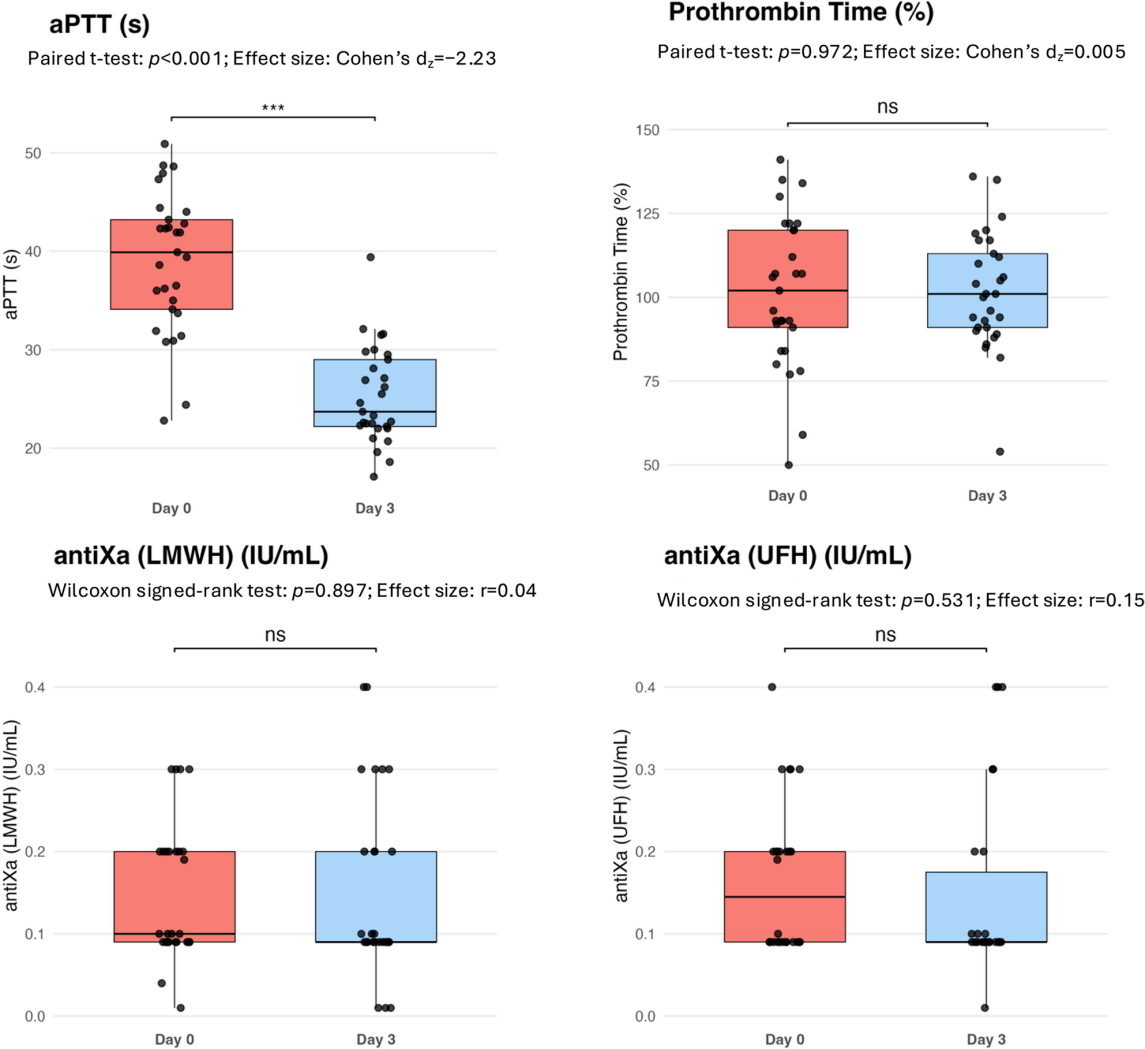
Changes in standard coagulation tests and anti-Xa activity after ECMO decannulation. Data are presented as boxplots, medians with interquartile ranges and individual patient values (dots). Effect sizes are reported as Cohen’s dz or rank-biserial correlation (r) depending on data distribution. aPTT, activated partial thromboplastin time; anti-Xa LMWH, anti-Xa activity calibrated for low-molecular-weight-heparin; anti-Xa UFH, anti-Xa activity calibrated for unfractionated heparin

**Fig. 3:**
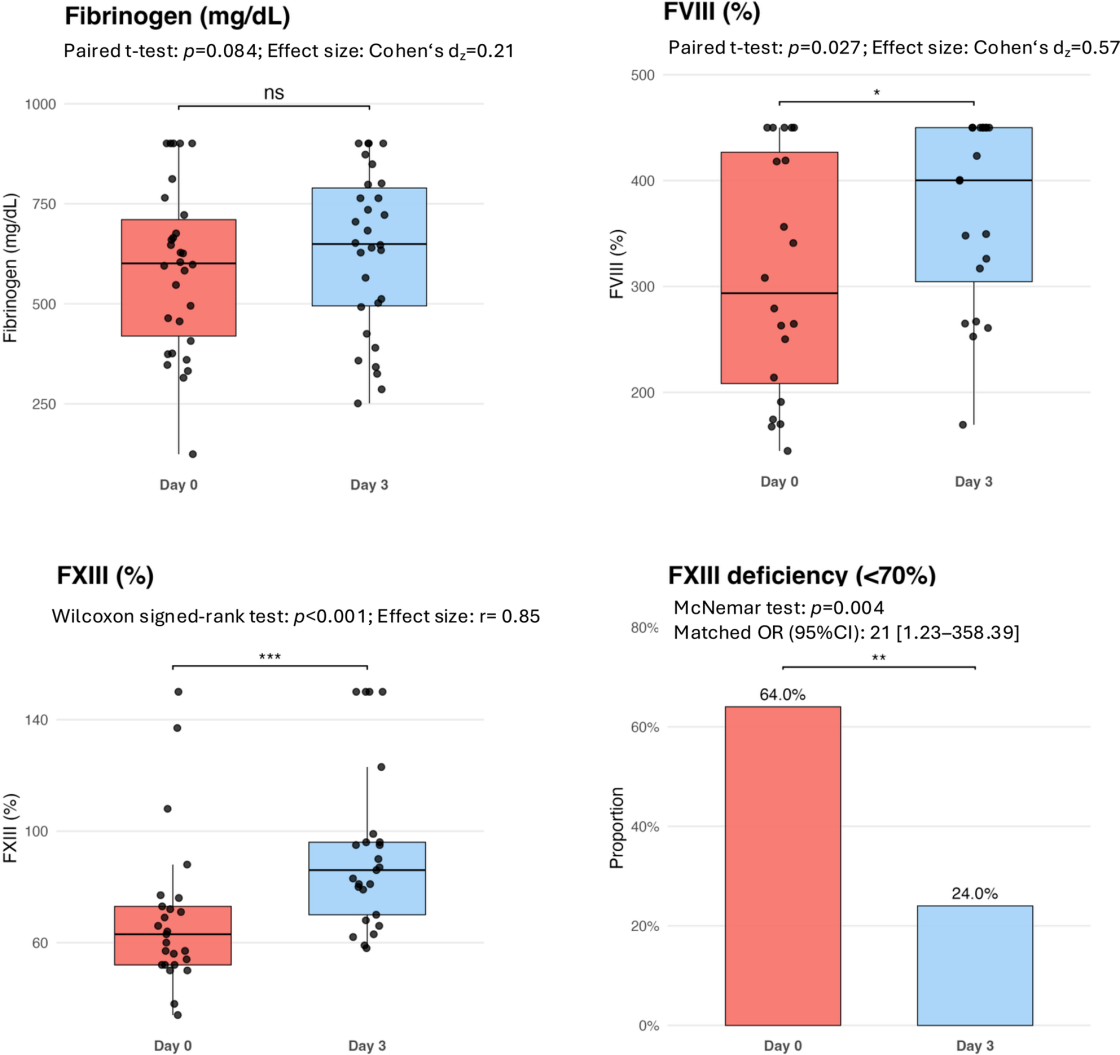
Changes in fibrinogen, Factor VIII, and Factor XIII following ECMO decannulation. Paired comparisons d0 and d3 illustrated by boxplots. Data are presented as medians with interquartile ranges and individual paired values. Bar graph illustrates proportion of patients with factor XIII deficiency (FXIII activity < 70%) at the two time points. Effect sizes are given as Cohen’s d_z_ or rank-biserial correlation (*r*), depending on data distribution

**Fig. 4:**
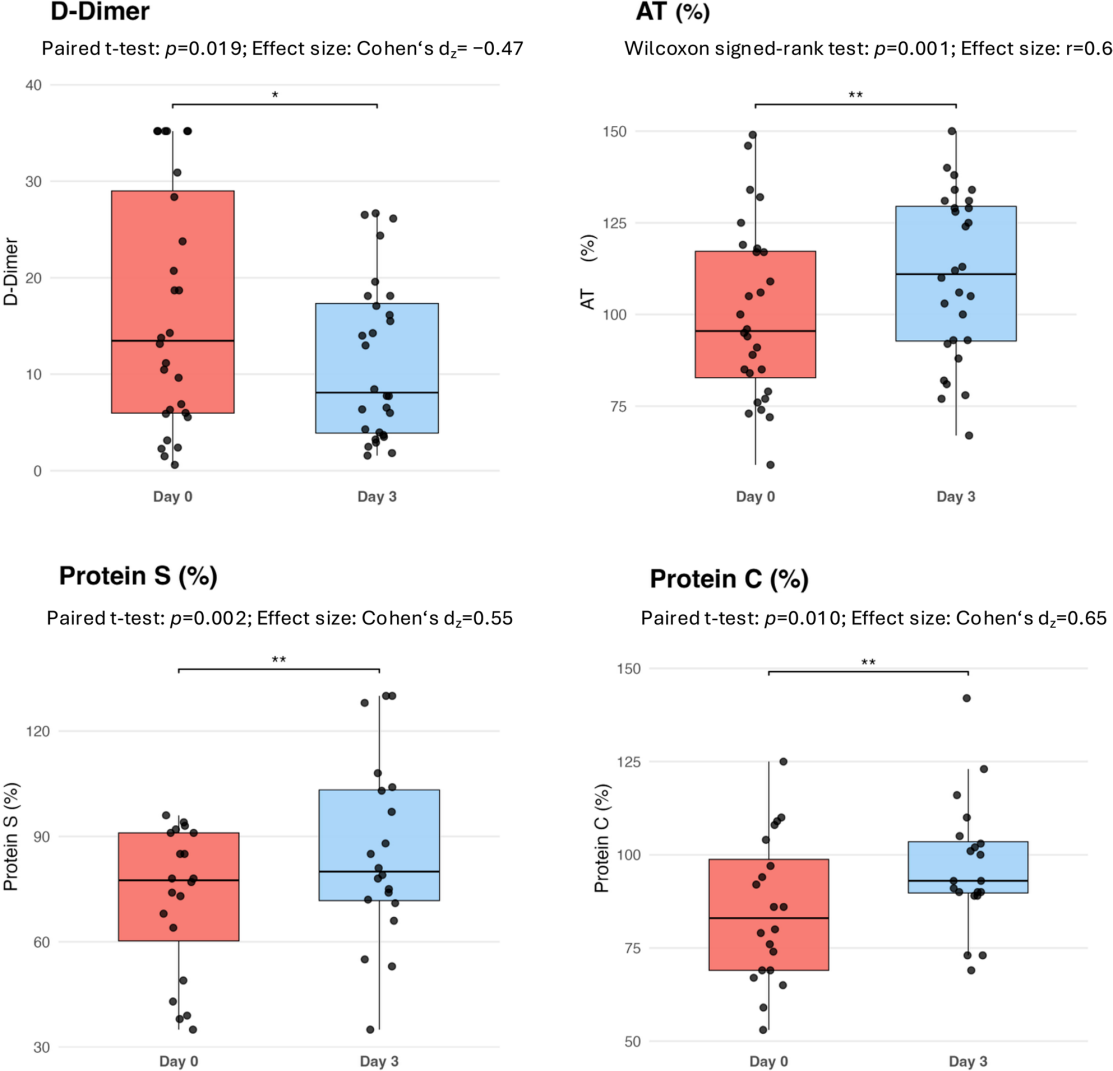
Changes in anticoagulatory proteins and D-dimer levels after ECMO decannulation. Data are presented as boxplots, medians with interquartile ranges and individual patient values (dots). Effect sizes are reported as Cohen’s dz or rank-biserial correlation (r) depending on data distribution.AT; antithrombin

Within 72 h after ECMO weaning and decannulation, platelet counts increased [97 (82–149) vs. 211 (161–346) G/L, *p* < 0.001)] and the proportion of thrombocytopenia declined significantly [22(73%) vs. 5(16.7%), *p* < 0.001] (Fig. 1). While the activated partial thromboplastin time significantly decreased from d0 to d3 [39.9 (32.20-53.35) vs. 23.7 (20.6–29.7) sec, *p* < 0.001], prothrombin time [102 (93.6-112.3) vs. 101 (91.8-110.5) %, *p* = 0.97] and anti-Xa levels calibrated for low-molecular-weight heparin [0.1 (0.09–0.18) vs. 0.09 (0.07–0.2) IU/ml, *p* = 0.90] and unfractionated heparin [0.15 (0.1–0.2) vs. 0.09 (0.07–0.22) IU/ml, *p* = 0.53] remained unchanged (Fig. 2). Only 1 study patient (3.3%) exhibited hypofibrinogenemia, and no patient was found to have Factor VIII deficiency at the time of decannulation. Fibrinogen levels trended upwards after decannulation [601 (419–711) vs. 650 (495–790) mg/dL, *p* = 0.08], without reaching significant differences between d0 and d3. While elevated Factor VIII activities increased further between d0 and d3 [294 (208–427) vs. 400 (304–450) %, *p* = 0.03], Factor XIII activities significantly increased from below to the normal range [63 (52–73) vs. 86 (70–96) %, *p* < 0.001]. The proportion of patients with Factor XIII deficiency significantly decreased from 16/25 (64%) to 6/25 (24%) study patients (*p* = 0.004) within 72 h after decannulation (Fig. 3). Stratification by time of exogenous Factor XIII exposure demonstrated median increases in FXIII activity between d0 and d3 in all groups, including Factor XIII administration only between d0 and d3 [ΔFXIII 19 (IQR 7–34.8) %], both before and after decannulation [ΔFXIII 29 (28–31) %], only prior to decannulation [ΔFXIII 15 (10–29) %], and no Factor XIII substitution [ΔFXIII 18 (8.5–55) %]. Activities of all anticoagulatory factors (AT, PC, PS) significantly increased between the two timepoints [AT: 96 (83–117) vs. 111 (93–130) %, *p* = 0.001; PC: 83 (69–99) vs. 93 (90–104) %, *p* = 0.01; PS: 78 (60–91) vs. 80 (72–103), *p* = 0.002]. D-Dimer levels significantly declined between d0 and d3 [13.5 (6–29) vs. 8.1 (3.9–17.3) %, *p* = 0.019] (Fig. 4). In the sensitivity analyses excluding patients, who received haemostatic products (platelet concentrates or fresh frozen plasma or coagulation factor concentrates) within 72 h following decannulation, trajectories of coagulation parameters pre-and post ECMO decannulation remained consistently similar (Electronic Supplementary Table 2).

### Changes in viscoelastic test results

Significant changes in the results of viscoelastic test results were observed within 72 h following decannulation. CT and CFT in the INTEM^®^ assay significantly decreased between d0 and d3. Both the clot amplitude at five and ten minutes, as well as the MCF in the INTEM^®^ and EXTEM^®^ assays significantly increased between the two timepoints. No changes were observed in the LI60 in any of the four ROTEM^®^ assays. Detailed results of viscoelastic tests are illustrated in Table 2.

**Table 2:** ROTEM^®^ coagulation markers before (d0) and at day 3 (d3) after ECMO decannulation

ROTEM^®^ parameters		d0	d3	relative change (d3 − d0)	Effect size^§^	*p*-value
APTEM^®^ CT	seconds	69 (58–78)	69 (66–79)	2.0 (−11.5–−5.5)	−0.05^#^	0.92
APTEM^®^ CFT	seconds	47 (41–56)	37.5 (35.5–60.5)	−5.5 (−11.5-2.0)	−0.45	0.32
APTEM^®^ A5	mm	53 (48–57)	60.5 (51.8–64.8)	6 (2.8–10.8)	0.90	0.08
APTEM^®^ A10	mm	63 (58–66)	69.5 (61.5–72.2)	5 (2.5–9.8)	0.87	0.09
APTEM^®^ MCF	mm	70 (66–74)	77.5 (69.5–78.8)	4.5 (3.0-7.5)	0.87	0.09
APTEM^®^ LI60	(%)	99 (97–100)	99 (99–100)	0 (0–0)	0.18	0.70
EXTEM^®^ CT	seconds	72 (66-78.5)	75 (67–79)	7 (−16 − 9)	−0.12	0.73
EXTEM^®^ CFT	seconds	50 (39.8–61.5)	39 (38–42)	−10 (−20 − −2)	−0.65	0.09
EXTEM^®^ A5	mm	54 (48–58)	63 (59–65)	11 (5–15)	1.02	0.02*
EXTEM^®^ A10	mm	64 (58.8–67.2)	72 (68–73)	10 (4–12)	0.91	0.03*
EXTEM^®^ MCF	mm	70.5 (65.5–73.2)	76 (72–79)	8 (3–9)	0.90	0.03*
EXTEM^®^ LI60	(%)	97 (96.5–99.2)	98.5 (96.8–99)	0 (−0.8 − 0.2)	−0.25	0.50
FIBTEM^®^ CT	seconds	74 (61.8–82)	78 (72–85)	3 (−7–13)	0.17	0.62
FIBTEM^®^ CFT	seconds	81.5 (47.5-227.2)	57 (46.5-168.5)	−30 (−452 − −16)	−0.93^#^	0.03*
FIBTEM^®^ A5	mm	24.5 (17.8–29.5)	26 (19–29)	3 (−1–3)	0.09	0.79
FIBTEM^®^ A10	mm	26.5 (19-31.8)	28 (21–30)	2 (−3–3)	0.0	1.0
FIBTEM^®^ MCF	mm	30 (21.8–36)	29 (24–34)	2 (−4 − 3)	−0.16	0.63
FIBTEM^®^ LI60	(%)	100 (100–100)	100 (100–100)	0 (0–0)	−1.00	0.32
INTEM^®^ CT	seconds	195 (169.8-224.5)	167 (162–179)	−30 (−60 − −7)	−1.09	0.01*
INTEM^®^ CFT	seconds	59 (44.2–69)	44 (38–48)	−21 (−32 − −9)	−0.87	0.02*
INTEM^®^ A5	mm	46.5 (44.8–52.2)	60 (55–60)	10 (7–16)	1.15	0.009*
INTEM^®^ A10	mm	57 (54.8–62.2)	68 (64–69)	9 (5–15)	0.98	0.02*
INTEM^®^ MCF	mm	64.5 (61.8–70.2)	73 (68–75)	7 (4–11)	0.99	0.02*
INTEM^®^ LI60	(%)	97 (95.8–100)	99 (98.5–99)	−0.5 (−1.0-1.2)	0.0	1.0

*CT* coagulation time, *CFT* clot formation time, *A5* clot amplitude at 5 min, *A10 *clot amplitude at 10 min, *MCF* maximum clot firmness, *LI60* lysis index at 60 min

^§^, effect sizes are presented as Cohen’s d_z_ or rank-biserial correlation (r) as indicated, depending on the distribution of data; ^#^, rank-biserial correlation (r) for non-normally distributed data (Wilcoxon signed-rank test);

*, significant change between d0 and d3

Data are given as median values with interquartile ranges in parentheses, if not otherwise indicated

## Discussion

In this exploratory, nested cohort study, we analysed changes in coagulation results during the first 72 h following ECMO weaning and decannulation. A consistent rise in platelet counts with a marked reduction in thrombocytopenia, a shortening of activated partial thromboplastin time, and significant increases in coagulation factor FVIII and FXIII activities were observed post decannulation. The natural anticoagulants AT, PC, and PS increased while D-dimer levels declined. Parallel improvements in viscoelastic parameters, such as shorter INTEM^®^ CTs and CFTs as well as higher MCFs, indicate restoration of clot initiation, propagation, and clot strength after decannulation. Taken together, these changes suggest a rapid restoration of haemostasis within the first 72 h following ECMO weaning and decannulation.

Our study explicitly investigated the early period after ECMO weaning, and its results align with those of a prospective study by Moerer et al., who reported spontaneous increases in clotting factors II, V, XI and XIII as well as platelet counts, fibrinogen and AT levels within 72 h after VV-ECMO discontinuation [24]. Furthermore, the results of our cohort including > 50% post-cardiotomy and eCPR patients corroborate such dynamics and extend the findings on post-decannulation coagulation dynamics to a broader patient population. Interestingly, FXIII activity showed an increase > 30% within the first 72 h after ECMO decannulation, corresponding to a 40% absolute reduction of the proportion of patients with FXIII deficiency (< 70%) from d0 to d3. The prevalence of acquired FXIII deficiency in ECMO patients is high and associated with bleeding and transfusion requirements [22, 29]. The observed rebound of FXIII and other coagulation factors suggests that ECMO-related blood trauma and consumption are likely to contribute to FXIII depletion, which is perceived to be a result of inflammation, underlying disease and circuit-related influences [21, 22]. Even though specific data for FXIII are lacking, an animal study on the use of ECMO in acute lung injury clearly demonstrated the degree of ECMO circuit-induced alterations on primary haemostasis, where initiation of extracorporeal circulation led to an early increase of collagen-induced platelet aggregation, a rapid decrease in FVIII and von Willebrand factor, and fibrinogen levels [33].

Additionally, the findings of our study provide some of the first systematic data on the course of anticoagulatory factors after ECMO termination, a field with limited prior evidence. The significant increases of AT, PC and PS levels within 72 h following decannulation suggest a rapid recovery from ECMO-related consumption and endothelial activation. Further, the observation that D-Dimer levels rapidly declined post-decannulation is in line with findings by Doyle et al. These authors demonstrated temporal changes in coagulation and fibrinolytic activation during ECMO therapy and within 24 h after ECMO removal in 17 VV-ECMO patients. While a decrease in D-Dimer and an increase in AT levels were detected on the first day after ECMO weaning, fibrinogen levels remained stable after decannulation [34].

We also evaluated changes in viscoelastic test results. Post decannulation changes detected by ROTEM^®^ remain poorly characterized. We detected a significant acceleration of thrombin-dependent endogenous clot initiation and formation/propagation parameters in INTEM^®^ assays. Additionally, an increase in clot strength in the recombinant tissue factor activated EXTEM^®^ assays was recorded. Lysis indices remained stable in all four assays, indicating absence of hyperfibrinolysis. However, interpretation of viscoelastic parameters requires careful consideration of their underlying determinants. Recent evidence suggests that CT fails to mirror thrombin elevation after 4 F-PCC spiking and is therefore less a direct marker of thrombin generation, but more strongly influenced by substrate availability, particularly fibrinogen [35, 36]. A recent in vitro study by Hofmann et al. demonstrated this dissociation between viscoelastic clot initiation parameters and thrombin generation on 4 different viscoelastic testing platforms, including ROTEM^®^, as thrombin level elevation after experimental haemodilution and 4 F-PCC spiking were not reflected by CT [37]. Accordingly, the observed shortening of CT after ECMO decannulation should be interpreted in the context of changes in coagulation factor availability (e.g., fibrinogen, FXIII) post decannulation, rather than as a direct surrogate of enhanced or improved thrombin generation. Similarly, CFT is closely linked to clot amplitude and MCF, and can be influenced by coagulation factor activity, but also platelet count, fibrinogen or haematocrit values [38]. Therefore, the observed increases in fibrinogen concentration or total platelet count post ECMO decannulation may have shortened CFT secondary to enhanced clot strength rather than faster thrombin generation per se.

Prior data on adult VV-ECMO suggest that discontinuation of extracorporeal exposure rapidly attenuates consumption of coagulation factors and platelets, potentially leading to a prothrombotic state [24]. Additional findings regarding hypercoagulatory shifts stem from paediatric longitudinal data that reported device-related consumption of factors XI and XII after ECMO initiation with significant recovery shortly after cessation of ECMO therapy [25]. Moreover, acquired von Willebrand syndrome has been shown to emerge rapidly within hours of cannulation and resolves promptly after decannulation, at times overshooting and creating a transient hypercoagulable state. This may imply an abrupt transition of primary haemostasis at the time of ECMO termination in both adult and paediatric patient cohorts [20, 26]. Similarly, the post ECMO decannulation period has been associated with a high incidence of post decannulation fever, and systemic inflammatory response syndrome (SIRS) patterns are frequently observed [39, 40]. Assouline et al. found that infections and thrombosis were observed in > 80% of cases after termination of ECMO support, raising the question if inflammatory response patterns might add to prothrombotic coagulation alterations in the critical post decannulation period [41].

The constellation of our study findings, an increase in both platelets and FXIII activity, combined with a marked shortening of aPTT, but parallel recovery of anticoagulatory proteins with increases in AT, PC and PS, are most consistent with an early restoration of haemostatic balance rather than an overshooting prothrombotic surge. However, FVIII activity demonstrated a significant increase to supraphysiologic levels in our cohort. The high FVIII activity levels in our cohort likely reflect systemic inflammation. As an acute-phase reactant, FVIII is released from endothelial cells in response to cytokines such as interleukin-6 [42]. Endothelial activation, commonly found in ECMO patients, promotes the exocytosis of Weibel–Palade bodies containing FVIII and von Willebrand factor [42–44]. Persistently high plasma levels may therefore indicate endothelial dysfunction and inflammatory activity rather than solely a reflection of enhanced coagulation status [45]. Nevertheless, elevated FVIII levels have been shown to increase the risk of both venous and arterial thrombosis via enhanced thrombin formation, induction of acquired resistance to activated PC, and increased platelet adhesion [46, 47]. Furthermore, post decannulation ROTEM^®^ results exhibited median EXTEM^®^ CFTs < 40 s, median EXTEM^®^ MCFs > 70 mm and median FIBTEM^®^ MCFs > 24 mm, all patterns compatible with the criteria of a hypercoagulable ROTEM^®^ profile [48].

### Limitations

Our study has several limitations which need to be considered. The sample size of 30 study patients was modest, reflecting the challenges associated with prospective sampling of critically ill ECMO patients. Since this was a single-centre study, the external validity and generalizability of our results are limited. Furthermore, the study cohort predominantly consisted of VA-ECMO patients. Therefore, its results may not equally apply to VV-ECMO, as VV- and VA-ECMO patients may differ in their coagulation profiles and thrombin generation [49]. Meaningful subgroup analyses comparing VA- and VV-ECMO were not feasible due to the small size of the VV-ECMO subgroup. Accordingly, potential differences in post-decannulation coagulation trajectories between ECMO configurations were not assessed. In addition, study patients received blood and coagulation products both during ECMO therapy and after ECMO decannulation, potentially influencing measurements of coagulation data. Although we performed sensitivity analyses to account for concomitant transfusion of blood products or administration of factor concentrates, residual confounding by other haemostatic therapies including anticoagulation or centre-specific treatment protocols cannot be ruled out. Nevertheless, stratified by the timing of FXIII substitution, increases in FXIII activity between d0 and d3 were observed across all exposure groups, including patients who did not receive FXIII either before or after decannulation, indicating that the post-decannulation rise in FXIII activity was not restricted to patients receiving exogenous FXIII. Fresh frozen plasma transfusions were administered in a small subset of patients in the context of perioperative bleeding during surgical ECMO decannulation. However, given the limited transfused volumes, fresh frozen plasma was unlikely to substantially increase individual coagulation factor activities, as previously demonstrated [50, 51]. Furthermore, we did not assess platelet function using formal platelet function testing (e.g., aggregometry) in our study. Therefore, interpretations and conclusions related to platelet recovery or a potential prothrombotic shift should be regarded as exploratory and hypothesis-generating, as platelet functional testing cannot be replaced by viscoelastic assays. Finally, data on the incidence or type of thromboembolic or haemorrhagic events during the observation period and beyond were not systematically collected.

## Conclusion

Extensive changes in coagulation profiles were observed within 72 h following ECMO weaning and decannulation. These changes reflect a rapid restoration of haemostasis and possibly an early shift towards a prothrombotic phenotype. 

## Supplementary Information

Supplementary Material 1.

Supplementary Material 2.

## Data Availability

The datasets generated and analysed during the current study are not publicly available, owing to the clinical nature of the dataset and restrictions by the Ethics Committee, but are available from the corresponding author on reasonable request.

## Abbreviations

A5: Clot amplitude at 10 minutes
A10: Clot amplitude at 10 minutes
aPTT: Activated partial thromboplastin time
AT: Antithrombin
CT: Clotting time
CFT: Clot formation time
ECMO: Extracorporeal membrane oxygenation
eCPR: Extracorporeal cardio-pulmonary resuscitation
ELSO: Extracorporeal Life Support Organization
4 F-PCC: Four-factor prothrombin complex concentrate
FXIII: Coagulation factor XIII
ICU: Intensive care unit
MCF: Maximum clot firmness
LI60: Lysis index at 60 minutes
LMWH: Low-molecular-weight-heparin
PC: Protein C
pRBC: Packed red blood cells
PS: Protein S
PT: Prothrombin time
ROTEM: Rotational thrombelastometry
SIRS: Systemic inflammatory response syndrome
UFH: Unfractionated heparin
VA: Veno-arterial
VV: Veno-venous
