# Supplementary material for: ECMO decannulation is associated with dynamic changes in coagulation profiles: an exploratory, nested cohort study
Source: BMC Anesthesiol. 2026 Jan 29;26:137. doi: 10.1186/s12871-026-03641-1 (PMC12924452; doi:10.1186/s12871-026-03641-1)
Supplement: Supplementary file 1 — Supplementary Material 1. [file 12871_2026_3641_MOESM1_ESM.docx]

**Electronic Supplementary Table S1.** Surgical and perioperative characteristics of patients undergoing surgery prior to ECMO initiation (n = 17)

| **Variable** |  | **Overall (all patients)** | **Recipients only** |
| --- | --- | --- | --- |
| **Duration of surgery** | min | 301 (188-412) | — |
| **Duration of CPB** | min | 211 (118-250) | — |
| **Type of surgery** |  |  |  |
| *Coronary artery bypass surgery* | n (%) | 6 (35.3) |  |
| *Mitral valve surgery/interventions* | n (%) | 5 (29.4) |  |
| *Aortic valve surgery/interventions* | n (%) | 2 (11.8) |  |
| *Tricuspid valve replacement* | n (%) | 2 (11.8) |  |
| *Aortic surgery (ascending aorta)* | n (%) | 1 (5.9) |  |
| *Abdominal surgery* | n (%) | 1 (5.9) |  |
| **Any RBC transfusion intraoperative** | n (%) | 8 (47.1) | — |
| **Any Platelet transfusion intraoperative** | n (%) | 6 (35.3) | — |
| **Any Plasma transfusion intraoperative** | n (%) | 5 (29.4) | — |
| **Number of RBC units transfused** | units | 0 (0-3) | 3.5 (2-4) |
| **Number of platelet units transfused** | units | 0 (0-1) | 1.5 (1-2) |
| **Number of plasma units transfused** | units | 0 (0-1) | 3 (2-3) |
| **4F-PCC dose intraoperative** | IU | 0 (0-1,800) | 1,900 (1,800-2,300) |
| **Fibrinogen dose intraoperative** | mg | 0 (0-4,000) | 4,000 (2,750-5,000) |
| **FXIII dose intraoperative** | IU | 0 (0-0) | not administered |
| **Tranexamic acid administered** | n (%) | 7 (41.2) | — |
| **Tranexamic acid dose** | mg | 5,553 (3,508-6,076.5) | — |
| **Aprotinin administered** | n (%) | 7 (41.2) | — |
| **Aprotinin dose** | IU | 2,929,200 (2,283,300-3,485,400) | — |
| **ECMO initiated in the operating room** | n (%) | 10 (58.8) | — |

CPB, cardiopulmonary bypass; ECMO, extracorporeal membrane oxygenation; 4F-PCC, Four-factor prothrombin complex concentrate; FXIII, coagulation factor XIII; IU, international units; RBC, red blood cells.

Data are given as median values with interquartile ranges in parentheses or n (%) if not otherwise indicated. “Recipients” refers to patients receiving the respective blood or coagulation product.
