# Supplementary material for: ECMO decannulation is associated with dynamic changes in coagulation profiles: an exploratory, nested cohort study
Source: BMC Anesthesiol. 2026 Jan 29;26:137. doi: 10.1186/s12871-026-03641-1 (PMC12924452; doi:10.1186/s12871-026-03641-1)
Supplement: Supplementary file 2 — Supplementary Material 2. [file 12871_2026_3641_MOESM2_ESM.docx]

**Electronic Supplementary Table S2.** Sensitivity analyses of paired changes in coagulation parameters between d0 and d3.

| **Variable** |  | **d0** | **d3** | **Δ d3-d0** | **Effect** | **Effect size^§^** | **p-value** |
| --- | --- | --- | --- | --- | --- | --- | --- |
| FVIII (%) | (%) | 294 (223-442) | 412 (279-450) | 51.6[−3.6-106.8] | Cohen's d_z_ | 0.49 | 0.07 |
| FXIII (%) | (%) | 66 (57-77) | 86 (80-123) | 20 [13-37.5] | r | 0.85 | <0.001* |
| Fibrinogen (mg/dL) | (mg/dL) | 615 (472-711) | 668 (518-800) | 48.6 [−2.8-99.9] | Cohen's d_z_ | 0.26 | 0.06 |
| Platelets (G/L) | (G/L) | 101 (86-175) | 212 (160-364) | 137.9 [87.9-188] | Cohen's d_z_ | 0.70 | <0.001* |
| Protein C (%) | (%) | 86 (70-102) | 93 (89-104) | 10.4 [0.9-19.9] | Cohen's d_z_ | 0.55 | 0.03* |
| Protein S (%) | (%) | 78 (69-91) | 83 (74-104) | 13 [4.3-21.7] | Cohen's d_z_ | 0.56 | 0.006* |
| AT (%) | (%) | 96 (85-118) | 112 (93-131) | 13 [6.5-18.5] | r | 0.58 | 0.004* |
| D-Dimer | μg/mL | 13.5 (6-29) | 8.1 (3.9-17.3) | −5.2 [−9.5-−0.9] | Cohen's d_z_ | −0.47 | 0.02* |
| aPTT | seconds | 40.9 (32.8-54.5) | 23 (20-28) | −14.2 [−17.8-−10.7] | Cohen’s d_z_ | −2.41 | <0.001* |
| PT | (%) | 104 (94-113) | 102.5 (92-110) | −0.08 [−5.9-5.7] | Cohen’s d_z_ | −0.004 | 0.98 |
| antiXa-LMWH | (IU/ml) | 0.1 (0.1-0.2) | 0.1 (0.07-0.2) | −0.03 [−0.11-0.06] | r | 0.18 | 0.57 |
| antiXa-UFH | (IU/ml) | 0.2 (0.1-0.3) | 0.1 (0.07-0.2) | −0.05 [−0.15-0.05] | r | 0.23 | 0.30 |

aPTT, activated partial thromboplastin time; AT, antithrombin; CI, confidence interval; FVIII, coagulation factor VIII; FXIII, coagulation factor XIII; LMWH; low-molecular-weight heparin; PT, prothrombin time; UFH, unfractionated heparin.

^§^, effect sizes are presented as Cohen’s d_z_ or rank-biserial correlation (r) as indicated, depending on the distribution of data; *, significant change between d0 and d3

Data are given as median values with interquartile ranges in parentheses if not otherwise indicated.
